# Reduce global cancer burden via cancer prevention and early detection of cancer

**DOI:** 10.1016/j.apjon.2022.03.011

**Published:** 2022-04-03

**Authors:** Marichen A. Dychangco

**Affiliations:** St. Paul University Manila, Advisory Board, Philippine Oncology Nurses' Association, Philippines

The last 30 years I have been reading, teaching, and advocating, what the World Health Organization says about cancer—that one-third of all cancers are preventable and one-third of all cancers are curable. If this saying were true, how come in 2018 there were an estimated 18.1 million new cancer cases globally and cancer remained the second leading cause of death,^1^ while there were 19.3 million new cases of cancer and almost 10 million deaths from cancer in 2020 and an estimated 28.4 million new cancer cases are projected to occur in 2040, a 47% increase from the corresponding 19.3 million cases in 2020.^2^ Where lies the problem?

Cancer burden refers to the estimated potential increase in the occurrence of cancer cases in a population subject to an incremental cancer risk of greater than one in one million resulting from exposure to toxic air contaminants. The estimates presented above paint a clear picture of the burden of cancer—it is escalating even though programs to prevent and detect cancer are present in every country. The burden of cancer is not just about statistics, the burden of being diagnosed with cancer, caring for a loved one with cancer, experiencing and witnessing pain, and letting go intrudes on the lives of almost every family around the world.

Yet as oncology nurses, we remain hopeful. We must stand firm in our quest to reach out to everyone because we believe that one-third of all cancers are preventable and one-third of all cancers are curable. And to do so, we must be more strategic in our pursuit to make everyone take an active part in preventing cancer occurrence and detecting cancer early. Since strategy is key, we must realize that the burden of cancer is not the same across the globe—between the developing and developed world, with particular cancer types exhibiting different patterns of distribution.

In 2012, 36.6% of cancer cases in high/very high Human Development Index (HDI) countries were associated with industrialized lifestyle factors and 9.6% to infections; while for low-medium HDI countries, 20.3% of cases were associated with lifestyle and 25% with infection related causes;^1^ however, in 2020, many countries classified with low and medium HDI levels are experiencing a marked increase in the prevalence of known cancer risk factors that prevail in high-income Western countries.^2^ What does this mean? A shift from infection-related and poverty-related cancers to lifestyle-related cancers is observed. This shift may affect cancer burden and may require a change in cancer prevention and control strategies.

This is not to say that existing cancer prevention programs that seek to reduce exposure to infectious or environmental are not important platforms, they still are, and will always be. But if the direction of cancer is towards our lifestyles—shouldn't our cancer prevention programs focus more on reducing behavioral risk factors and modeling them as nurses? Programs to make people understand the importance of having a normal body mass index, the need to include fruits and vegetables in the daily diet, the benefits of doing regular exercise, the wonders smoking cessation and alcohol consumption moderation does to the body.^3^ And at the center of all these cancer prevention programs must be an authentic advocate—a nurse who models a healthy lifestyle. Nurses must lead in the programs on cancer prevention and early detection.

Early detection of cancer continues to be a vital preventative intervention. Methods for supporting early identification include improving public awareness, case finding and screening. Cancer screening program success is depended on three common principles. First, there needs to be an available test procedure that is acceptable, safe, and relatively inexpensive. Second, the cancer should be one that is associated with high morbidity and mortality in that area so people will realize the relevance. The burden of cancer in the world varies according to the community and programs must be targeted and significant. And third, funding for effective screening, diagnosis and treatment needs to be available.^3^

In the now normal, when the focus is on the pandemic, economic instability, and conflicts between and among nations—the burden of cancer remains. Let us not rest on our quest for the prevention and early detection of cancer. Evidence still suggests that multiple approaches at any given time are still more effective. And these include behavioral strategies, commitment, leadership, and collaboration with local and national institutions for funding and policy implementation.

Be it at the individual, institution (school or hospital), community or government levels, let us remember that we are all responsible in preventing and early detection of cancer. Let us take the initiative to act and let us do it now. My own contribution is this motto: CEEASE the Big C. **C**ease smoking, **E**ducate everyone and anytime, **E**mpower all nurses, **A**void unnecessary exposures, **S**tay health in all dimensions, and let us push for **E**arly testing and screening.

## Declaration of competing interest

None declared.
